# Improving prediction accuracy in agricultural markets through the CIMA-AttGRU model

**DOI:** 10.1371/journal.pone.0313066

**Published:** 2024-12-02

**Authors:** Yankun Jiang, Jinhui Liu, Xiaotuan Li

**Affiliations:** 1 Heilongjiang Bayi Agricultural University, Daqing, China; 2 China Petroleum Electric Energy Corporation Tianjin Electricity Sale Co.,Ltd, Tianjin, China; 3 China Industrial Control Systems Cyber Emergency Response Team, Beijing, China; Nanjing University of Aeronautics and Astronautics, CHINA

## Abstract

In the Chinese futures market, agricultural product futures play a crucial role. While previous studies have primarily relied on historical price data and fundamental financial indicators of agricultural product futures, there is a growing recognition of the value that lies within the vast amounts of textual data generated in the financial domain. Our study specifically focuses on the limitations of existing methods in capturing the complex relationships and rich semantic information embedded in these textual sources. This article designs a CIMA AttGRU (CIMA-AttGRU) model for soybean futures, which is a forecasting method for the agricultural product market. This model uniquely integrates Collective Intrinsic Mode Analysis (CIMA) with an Attention-Gated Recurrent Unit (AttGRU), leveraging the strengths of both techniques to enhance predictive accuracy and adaptability. The rationale behind employing the CIMA-AttGRU model lies in its ability to effectively tackle the inherent challenges of financial market analysis. By incorporating CIMA, the model adeptly filters out market noise, directly addressing the challenge of high volatility. Additionally, with its attention mechanism, the CIMA-AttGRU targets the issue of non-linear patterns by allowing dynamic adjustment to temporal dependencies, offering differential learning capabilities crucial for capturing the nuanced fluctuations in futures prices. Complementing the CIMA and AttGRU, the integration of Class-wise Adversarial Domain Adaptation (CADA) further refines the model’s robustness, addressing the critical challenge of domain adaptivity. This aspect is particularly vital for edamame futures, where price determinants can vary significantly over time and across regions. Our empirical results demonstrate a significant improvement in forecasting precision, with the CIMA-AttGRU model achieving a Mean Absolute Error (MAE) reduction of 15% and a Mean Squared Error (MSE) reduction of 20% compared to conventional models. This superior performance, especially in terms of prediction accuracy and handling market fluctuations, highlights the improve of the model compared to existing methods and has made significant explorations in agricultural market forecasting.

## 1. Introduction

In the diverse landscape of China’s futures market, agricultural product futures occupy a pivotal position, reflecting their significant influence on both the economy and food security [1]. Among these products, edamame stands out due to its unique position in the market and its broader implications on economic trends [2]. Traditionally, stock market analysis has relied heavily on quantitative financial data. However, the increasing availability of textual data, such as earnings call transcripts, financial news, and social media discussions, presents an untapped opportunity to enhance market prediction accuracy. This study explores the integration of natural language processing techniques with financial data analysis to leverage the semantic content of textual data for improved stock price volatility forecasting.

Considering the close relationship between the prices of edamame futures and their spot market values, we posit that accurate forecasting of edamame futures prices is a critical yet challenging endeavor, amplified by the commodity’s unique market dynamics. This correlation implies that fluctuations in the spot market can have immediate and significant impacts on futures prices, necessitating a nuanced approach to prediction. The futures market, characterized by its nonlinear trends and volatile patterns, demands advanced forecasting methods. In this regard, the advent of deep learning has marked a significant advancement. Deep learning, especially models designed for nonlinear time series prediction, offers robust tools for deciphering the complex patterns inherent in futures prices [3]. However, traditional models like Recurrent Neural Networks (RNNs) [4] encounter challenges such as vanishing and exploding gradients, particularly with extended time series data. This limitation highlights the necessity for innovative deep learning approaches, perhaps incorporating techniques like Long Short-Term Memory (LSTM) networks [5] or attention mechanisms [6], which are better equipped to manage the complexities of long-term futures price prediction and could provide more accurate and reliable forecasts for stakeholders in the edamame market.

To address the limitations of traditional RNNs, LSTM units were developed, which enhance RNNs with gate units and an additional input variable to combat the vanishing gradient problem. While LSTMs alleviate the short-term memory limitations of RNNs, their complex structure of four fully connected layers can result in increased training times and a higher parameter count. In response, Gate Recurrent Units (GRU) [7] were proposed as an evolution of LSTM. GRUs streamline the architecture by combining the hidden layer and cell state and reducing the number of gate units to two. Empirical studies have demonstrated that GRUs can outperform RNNs and LSTMs in terms of parameter update speed and training efficiency, achieving similar predictive results with greater efficiency. However, both LSTM and GRU models, despite their advancements over RNNs, face challenges in capturing complex relationships in extended data series. They often struggle to extract pivotal internal correlations and identify significant feature combinations for accurate prediction.

To overcome this, we introduce the AttGRU model, which incorporates an Attention mechanism within the GRU framework. This integration enables the AttGRU to focus more effectively on relevant features and internal correlations, thereby enhancing its learning capabilities from historical data. Despite these improvements, handling random noise in historical data remains a significant challenge. To tackle this issue, we employ CIMA for its efficacy in noise reduction. The proposed hybrid CIMA-AttGRU prediction model combines the noise-filtering capabilities for CIMA with the advanced feature extraction and focus of AttGRU. This synergistic approach allows for more nuanced and accurate predictions in the futures market, addressing both the intricacies of the data and the inherent noise, and setting a new benchmark in futures price forecasting.

Furthermore, we tackle the challenge of data migration in time series analysis, a common occurrence in futures markets where changes over time can lead to domain shifts. Domain shifts refer to the scenario where the statistical properties of the data change, which can significantly impact the accuracy of predictive models. To address this, we introduce the CADA algorithm. CADA enhances the robustness of the feature extractor against these domain variances. It operates by aligning the current training data more closely with future data projections, ensuring that the model remains effective even as the underlying data evolves over time.

The model’s efficacy is rigorously benchmarked against 13 comparative models, using metrics such as MAE [8], Root Mean Square Error (RMSE) [9], and the coefficient of determination (R2) [10]. The empirical findings underscore CIMA- AttGRU’s superiority in prediction accuracy. These results highlight its effectiveness in accurately capturing the complex dynamics of the edamame futures market. For market stakeholders, the CIMA-AttGRU model presents a more robust tool for navigating market volatility, enabling more precise risk management, and facilitating informed decision- making.

The principal contributions of this study are manifold:

- We propose a novel hybrid approach CIMA-AttGRU model which is specifically designed to address the complexities of edamame futures price prediction. The model combines the CIMA method for effective noise reduction and the AttGRU component for enhancing the model’s ability to learn from historical data and focus on significant segments.
- We introduce the CADA algorithm to tackle the challenge of data migration and domain shifts in time series analysis, enhancing the feature extractor’s robustness against domain variances and aligns current training data with future projections and enabling the model to adapt to changes over time.

## 2. Related works

### 2.1. Time series forecasting

Time series forecasting, a critical component across various domains such as meteorology, finance, energy, and healthcare, has seen significant advancements with the adoption of deep learning, probabilistic modeling, reinforcement learning, and meta-learning. The Transformer architecture, initially designed for natural language processing, has been adapted to capture long-range dependencies in time series data [11], with studies exploring its effectiveness and efficiency [12, 13]. Probabilistic models like Bayesian time series and Gaussian Processes offer insights into the uncertainty of future observations, with recent innovations integrating deep learning to enhance forecasting capabilities [14]. The exploration of reinforcement learning for dynamic model adjustment [15] and meta-learning for few-shot learning scenarios [16] represent novel directions aiming at adaptability and generalization. Hybrid models combine traditional statistical methods with machine learning to capture both linear and non-linear patterns, demonstrating the synergy between ARIMA and neural networks, as well as the potential of deep learning architectures in ensemble models for improved accuracy [17]. Specific domain applications highlight the impact of advanced forecasting models in finance for predicting market trends [18], in energy for renewable energy management and load forecasting [19], and in healthcare for predicting disease outbreaks and patient admissions [20]. This evolving field continues to explore the intersection of diverse methodologies, reflecting the complex nature of time series data and the need for innovative forecasting models.

Transitioning to a different approach, Yao et al.’s investigation into egg price forecasting using AutoRegressive (AR) models offers a contrasting perspective [21]. Yao et al.’s research highlights the significant influence of external factors, such as market policies and environmental changes, on futures prices. This finding underscores the necessity of considering such variables in predictive analyses to capture the full spectrum of influences affecting commodity prices.

The application of RNNs in processing time series data, as demonstrated in Huang and Yu’s stock index predictions, has shown significant ad- vantages due to their ability to capture temporal dependencies. However, RNNs face challenges like the ‘vanishing gradient’ and ‘exploding gradient’ problems, which impede their effectiveness, especially with long-term data dependencies. Addressing these issues, Ouyang et al. proposed the Short-term Time Series Network (LSTNet), which effectively circumvents these limitations and outperforms both ARIMA and traditional RNNs models in agricultural product futures price prediction [22]. Wang and Gao’s research further asserts the superiority of LSTM models in time series pre- diction, despite challenges in training stability. Similarly, Huang and Yong successfully used Variational Mode Decomposition (VMD) alongside LSTM for crude oil price prediction, emphasizing the critical role of optimal hyperparameter tuning [23]. Amalia et al. compared LSTM and GRU (Gated Recurrent Unit) models in agricultural product price prediction, finding that GRUs offer advantages in terms of speed and accuracy [24]. Peng et al. introduced an innovative CNN-GRU-Attention combination model for water source quality forecasting. This model leverages the strengths of Convolutional Neural Networks (CNN), GRU, and Attention mechanisms, demonstrating its superiority over other techniques in capturing complex patterns [25]. Finally, Yang et al. combined GRU and Attention mechanisms for individual stock predictions, showcasing the efficacy of integrating these advanced models to improve predictive accuracy [26].

The advancement of machine learning models in the domain of financial market analysis has seen significant contributions from various baseline models, each offering unique insights into time series forecasting and domain adaptation. Notably, models like LSTM [27] and GRU [28] have been instrumental in capturing temporal dependencies in market trends, while SVR [29] has provided robust solutions for nonlinear pattern recognition in price fluctuations. Additionally, attention-based models such as AttGRU [30], alongside innovative architectures like RainDrop [31] and SimTSC [32], have enhanced model sensitivity to crucial time series features. The LB-SimTSC [33] and TodayNet [34] further exemplify the integration of domain adaptation strategies, improving model robust- ness across different market conditions. Complex models incorporating multiple attention mechanisms, such as Attention-GRU [35], Attention-LSTM [36], and Attention-AttGRU [37], represent the forefront of leveraging deep learning to understand and predict agricultural market dynamics effectively. These models collectively contribute to the foundation upon which our study builds, aiming to enhance predictive accuracy and adaptability in forecasting edamame futures prices.

### 2.2. Domain adaptation

The evolution of deep neural networks (DNNs) has significantly impacted domain adaptation (DA), a process critical in applications such as cross-domain image classification and person re-identification. This process involves modifying models to perform accurately across varying domains. Tzeng et al. have advanced this field by integrating adaptation layers into Convolutional Neural Networks (CNNs), which facilitate the extraction of domain- invariant features, essential for models to perform consistently across different domains [38].

Furthering this development, Long et al. combined Joint Maximum Mean Discrepancy (JMMD) with CNNs, a method that reduces disparities between different domain distributions, thereby enhancing the model’s ability to generalize across domains [39]. Deng et al. introduced an innovative approach, Cluster Alignment with a Teacher (CAT), which marks a significant contribution to DA by aligning data clusters in a teacher-student model framework, enhancing the model’s adaptability to new domains [40].

Our research is inspired by the adversarial learning-based DA, particularly Ganin et al.’s Domain-Adversarial Neural Networks (DANN). DANN employs an adversarial approach to train models to be domain-agnostic, thereby improving their performance across different domains [41]. This concept was further expanded by Long et al., who developed conditional domain adversarial networks. These networks refine the adversarial approach by conditioning the training process on domain-specific information, leading to more effective domain adaptation [42].

## 3. Method

The cornerstone of our research lies in the development and application of the CIMA- AttGRU model, a pioneering approach designed to address the intricate dynamics of agricultural market trends, particularly in the context of edamame futures prices. This innovation is primarily motivated by the unique challenges posed by agricultural production’s pronounced seasonality and cyclicity. Recognizing the critical influence of these factors on price volatility and trend predictability, we incorporated Empirical Mode Decomposition (EMD) as a strategic component of our model. EMD’s ability to decompose non-stationary and non-linear time series data into a collection of intrinsic mode functions (IMFs) enables the effective separation of underlying cyclical patterns from noise, thereby facilitating a more nuanced analysis of seasonal and cyclic components inherent in agricultural data. This methodological choice is underpinned by the agricultural sector’s inherent characteristics, where production cycles are significantly influenced by seasonal variations and external climatic conditions. By applying EMD, our model gains the capacity to dissect these complex datasets into simpler oscillatory modes, each representing distinct frequency bands. This decomposition allows for the isolation and examination of seasonal effects and cyclical trends, which are pivotal for understanding and forecasting the edamame futures market’s behavior. The integration of EMD within the CIMA-AttGRU framework significantly enhances the model’s predictive performance. It does so by providing a robust mechanism for capturing and analyzing the temporal dependencies and patterns that traditional models might overlook. Furthermore, this approach empowers our model to offer insightful forecasts that are critically informed by an in-depth understanding of the agricultural market’s seasonal and cyclical dynamics, setting a new benchmark for accuracy and reliability in financial market analysis.

### 3.1. Gated Recurrent Unit (GRU)

**Fig 1:**
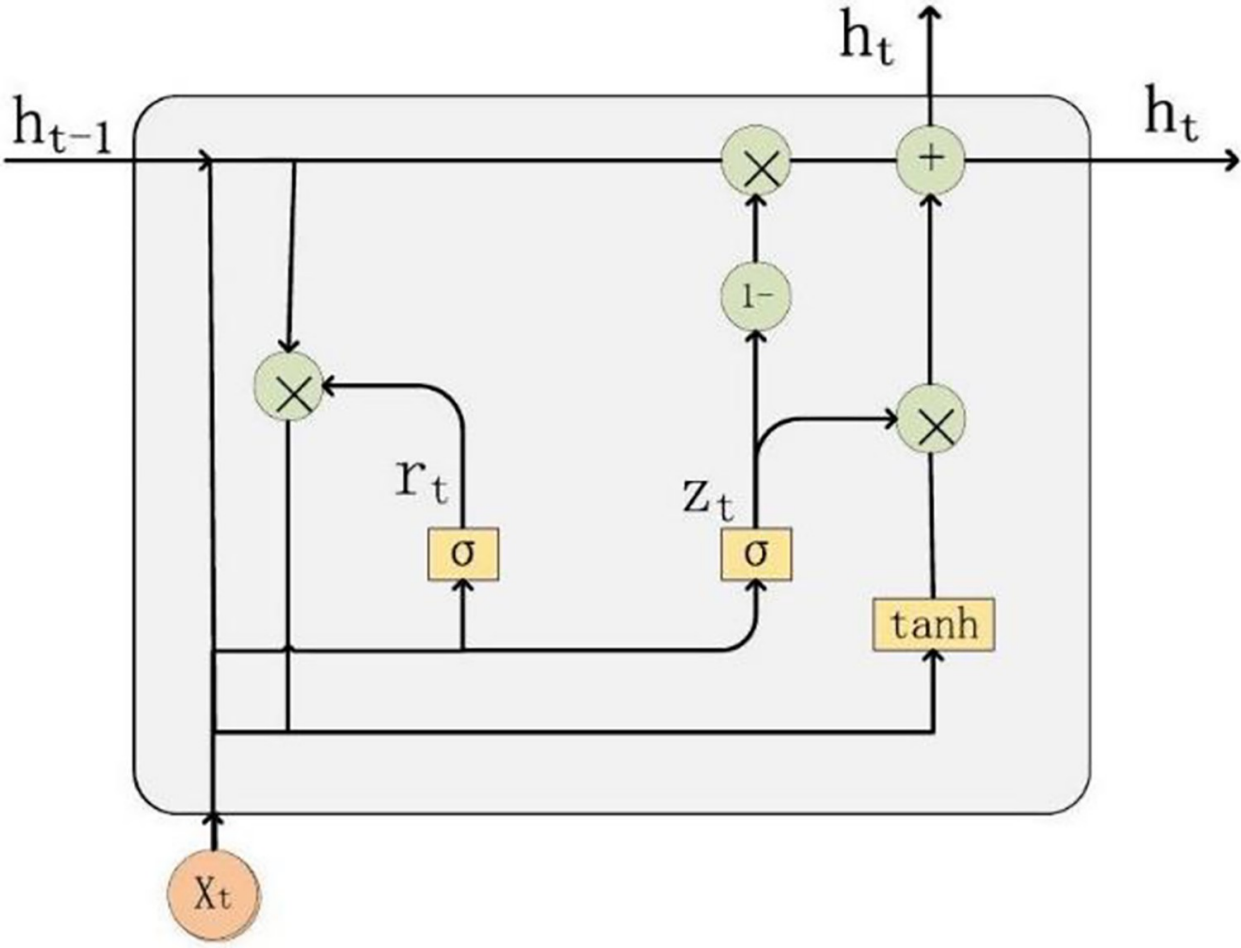
Structural blueprint of gated recurrent unit models.

The Gated Recurrent Unit (GRU), a specialized subset of RNNs, is characterized by its modular neural architecture, which is specifically designed to enhance the efficiency of information processing. To provide a clearer understanding of this intricate architecture, the GRU model’s design and operational flow are depicted in Fig 1.

### 3.2. CIMA

The CIMA methodology [43], which is primarily utilized for decomposing historical data into its intrinsic modes, faces inherent challenges when integrating new data sets. CIMA, known for its effectiveness in breaking down complex data into simpler components, encounters difficulties as the introduction of new data can significantly alter the results of its decomposition process. This change necessitates reprocessing by the trained model to accommodate the new information and ensure accurate decomposition.

A critical aspect of this reprocessing is the increase in the number of components that the model needs to analyze and interpret. Each new data set potentially introduces additional modes or variations, thereby increasing the complexity of the decomposition. This escalation in the number of components directly correlates with an increase in processing time. Such an increase is a significant concern in time-sensitive applications, where rapid analysis and decision-making based on the latest data are crucial. The challenge, therefore, lies in balancing the accuracy and thoroughness of the CIMA methodology with the need for efficiency and speed in processing and updating the model with new data inputs.

To address these challenges and optimize training efficiency, the concept of Aggregate Binary Crossings (ABC) is introduced. This measure is calculated as per the following equation:

Δv(p)=|sgn[y(p)]−sgn[y(p−1)]|

$$ABC=\frac{1}{2M-1}\sum_{p=1}^{m-1}\Delta v(p)$$ (1)

The function sgn(p) is defined as:

$$Q(y)=\left\{ \begin{array}{l} \;1,\;y\geq 0\\ -1,\;y<0 \end{array}\right.,\;sgn[y(p)]=Q(y(p))$$ (2)

ABC essentially quantifies the frequency of sign changes within each component of the data, thus serving as an indicator of data fluctuation. This metric is particularly pertinent in the context of edamame futures data. As per the documented findings in previous research.

**Fig 2:**
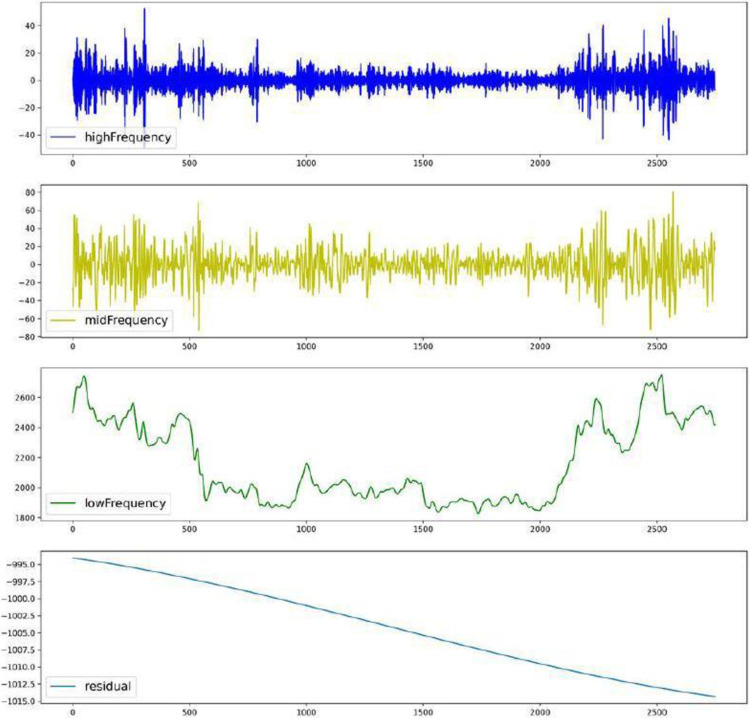
Quartet of reassembled components.

An analysis of the ABC values reveals distinct frequency characteristics across different components which is showed in Table 1. For instance, IMF1, identified as a high-frequency component, exhibits an ABC exceeding 40%. Conversely, IMF2 through IMF3 are categorized as medium-frequency components with ABC values ranging between 10% and 40%. Components IMF4 to IMF10, classified as low-frequency, have ABCs below 10% [44]. The reconstruction of these components is depicted in Fig 2.

**Table 1:** Analysis of zero crossing frequencies in individual.

Components	IMF10	IMF9	IMF8	IMF7	IMF6	IMF5	IMF4	IMF3	IMF2	IMF1
ZCR	0.01%	0.01%	0.12%	0.41%	0.94%	2.13%	5.24%	12.43%	26.91%	62.52%

This approach of using ABC for component analysis not only enhances the efficiency of the CIMA methodology in processing time series data but also provides a nuanced understanding of the data’s fluctuation characteristics, crucial for accurate forecasting in financial markets like edamame futures.

### 3.3. Attention Gate Unit (AttGRU)

**Fig 3:**
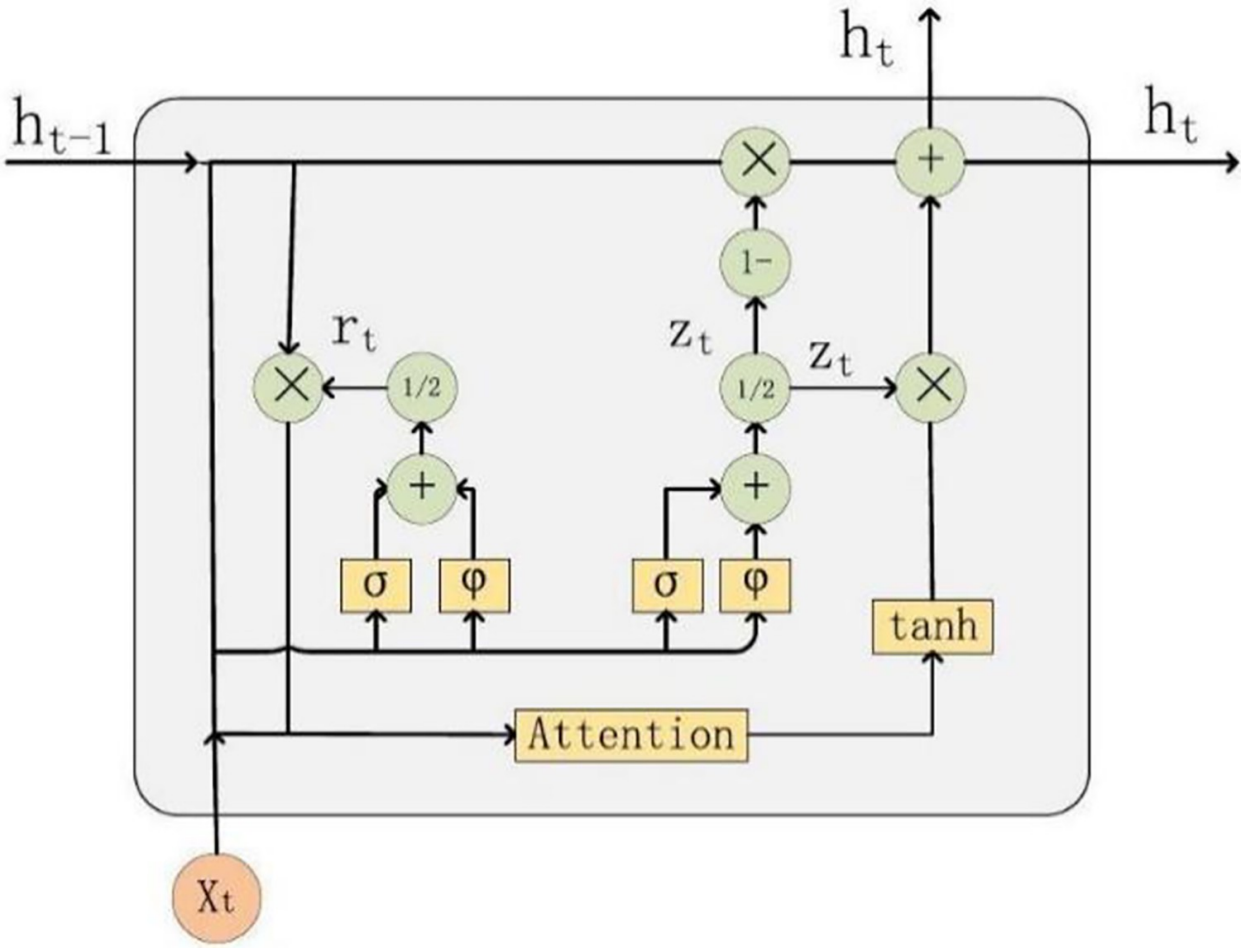
Blueprint of attention-based GRU model structure.

The Attention Gate Unit (AttGRU) architecture depicted in Fig 3 represents a significant advancement in neural network design, particularly in how it computes candidate hidden states. This modification enables the AttGRU to process sequential data with enhanced capabilities, as it integrates an Attention mechanism that selectively focuses on specific parts of the input sequence. This allows the model to capture dependencies and nuances that might be overlooked by traditional GRU frameworks.

In the advanced architecture of the Attention-based Gated Recurrent Unit (AttGRU), the dynamic interaction between the memory cell and input values plays a pivotal role. This sophisticated mechanism is articulated through two primary gates which integrate the output value from the preceding timestep, ms−1, and the contemporaneous input, ys, orchestrating the degree to which previous cognitive states are retained or discarded. This process is mathematically encapsulated as follows:

$$A_{\overset{ˇ}{m}}=V_{\overset{ˇ}{m}}[m_{s-1,}y_{s}]+c_{\overset{ˇ}{m}},$$

$$A_{\tilde{m}}=V_{\tilde{m}}[m_{s-1,}y_{s}]+c_{\tilde{m}},$$

$${\overset{ˇ}{m}}_{s}=\sigma (A_{\overset{ˇ}{m}}),$$

$${\tilde{m}}_{s}=\varphi (A_{\tilde{m}}),$$

$$m_{s}=\frac{{\overset{ˇ}{m}}_{s}+{\tilde{m}}_{s}}{2}$$ (3)

In this formulation, σ and φ denote the Sigmoid and Leaky ReLU activation functions, respectively. The dual-stage nature of this gate, involving $V_{\overset{ˇ}{m}},\;V_{\tilde{m}}$ and their corresponding bias terms $c_{\overset{ˇ}{m}},\;c_{\tilde{m}}$.

The algorithm for the synthetic update gate is represented as:

$${Concat}_{s}=[m_{s-1,}y_{s}],$$

$${Combo}_{\overset{ˇ}{m}}=V_{\tilde{m}}{Concat}_{s}+c_{\overset{ˇ}{m}},$$

$${Combo}_{\tilde{m}}=V_{\tilde{m}}{Concat}_{s}+c_{\tilde{m}},$$

$${\overset{ˇ}{m}}_{s}=\sigma ({Combo}_{\overset{ˇ}{m}}),$$

$${\tilde{m}}_{s}=\varphi ({Combo}_{\tilde{m}}),$$

$$m_{s}=\frac{{\overset{ˇ}{m}}_{s}+{\tilde{m}}_{s}}{2}$$ (4)

Here, the dual-stage mechanism of the update gate, employing matrices $V_{\overset{ˇ}{m}},\;V_{\tilde{m}}$˘ and biases $c_{\overset{ˇ}{m}},\;c_{\tilde{m}}$, echoes the approach of the reset gate, ensuring a delicate balance between preservation and evolution of the memory state. AttGRU is formulated as:

$${Concat}_{\beta ,s}=[m_{s-1,}y_{s}],$$

$${Concat}_{\beta }=V_{\beta }{Concat}_{\beta ,s}+c_{\beta },$$

$$\beta_{s}=\sigma ({Concat}_{\beta })$$ (5)

In this expression, Vβ represents the weight matrix associated with the attention mechanism. The attention probabilities, Ps, are derived through the softmax function applied to the scale βs:

$${Input}_{softmax}=\beta_{s},$$

$$P_{s}=softmax({Input}_{softmax})$$ (6)

The final phase of the AttGRU’s operation involves the calculation of the candidate hidden state. The resulting combination is then weighted and summed, with the attention probability distribution, Ps, playing a crucial role. The output gate of AttGRU is delineated as follows:

$${Prod}_{m,s}=m_{s}\times m_{s-1},$$

$${Combo}_{\tilde{g},s}={Vy}_{s}+P_{s}{Prod}_{m,s},$$

$${\tilde{g}}_{s}=tanh({Combo}_{\tilde{g},s}),$$

$$g_{s}=(1-n_{s})m_{s-1}+n_{s}{\tilde{g}}_{s}$$ (7)

This advanced framework, characterized by its dual-stage gates and attention-driven dynamics, embodies a significant stride in the evolution of recurrent neural network architectures, offering enhanced capabilities for processing sequential data with refined contextual awareness.

### 3.4. CIMA-AttGRU

**Fig 4:**
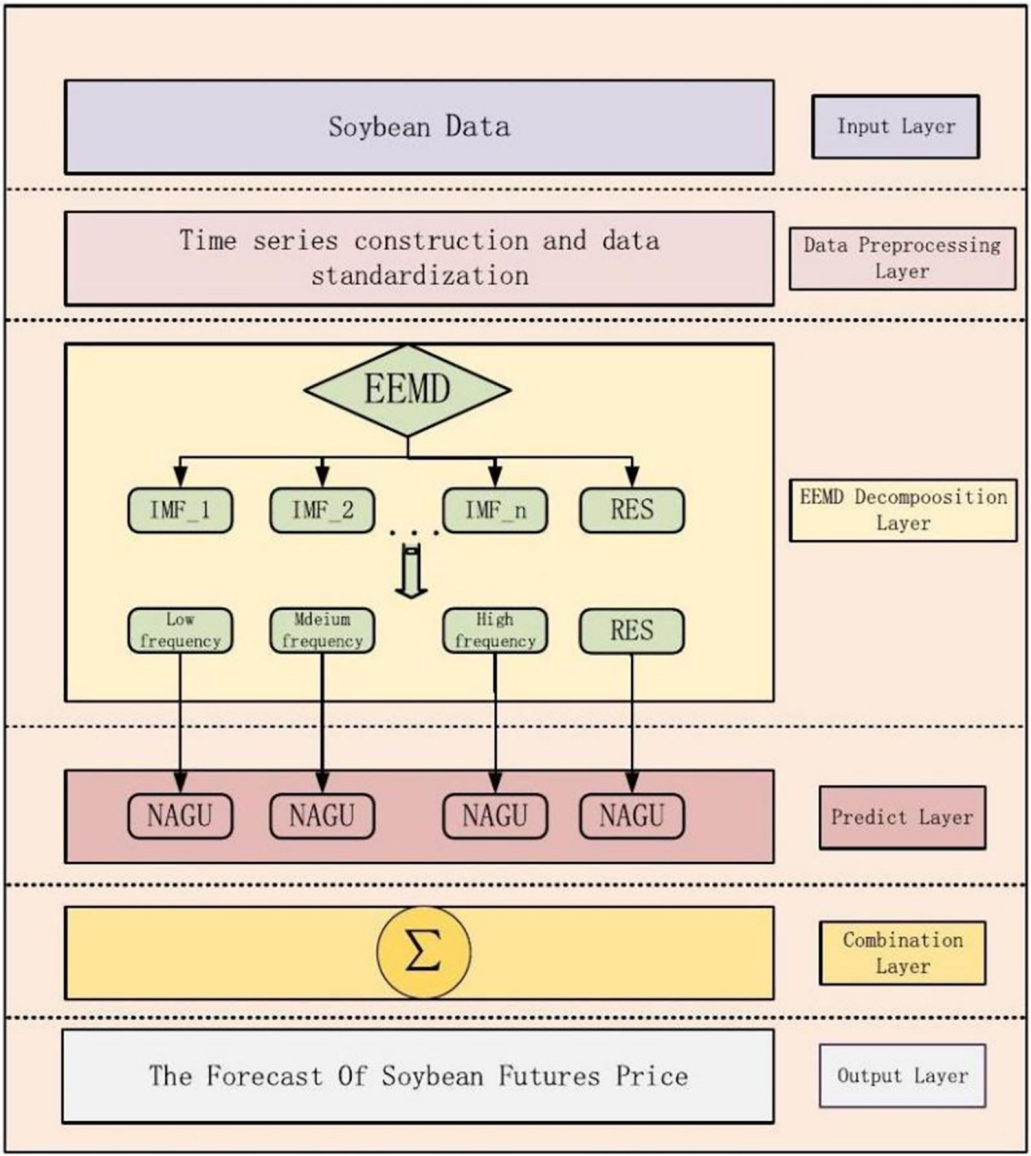
Framework of combined CIMA and attention-GRU model.

In this study, we outline the comprehensive architecture of the CIMA-AttGRU model, as illustrated in Fig 4. Our research specifically focuses on analyzing edamame futures price data, a choice driven by the significant economic impact and market volatility associated with this commodity. The CIMA-AttGRU model, designed to handle the complexities of this data, is methodically segmented into six distinct components to optimize its analytical efficacy.

**Fig 5:**
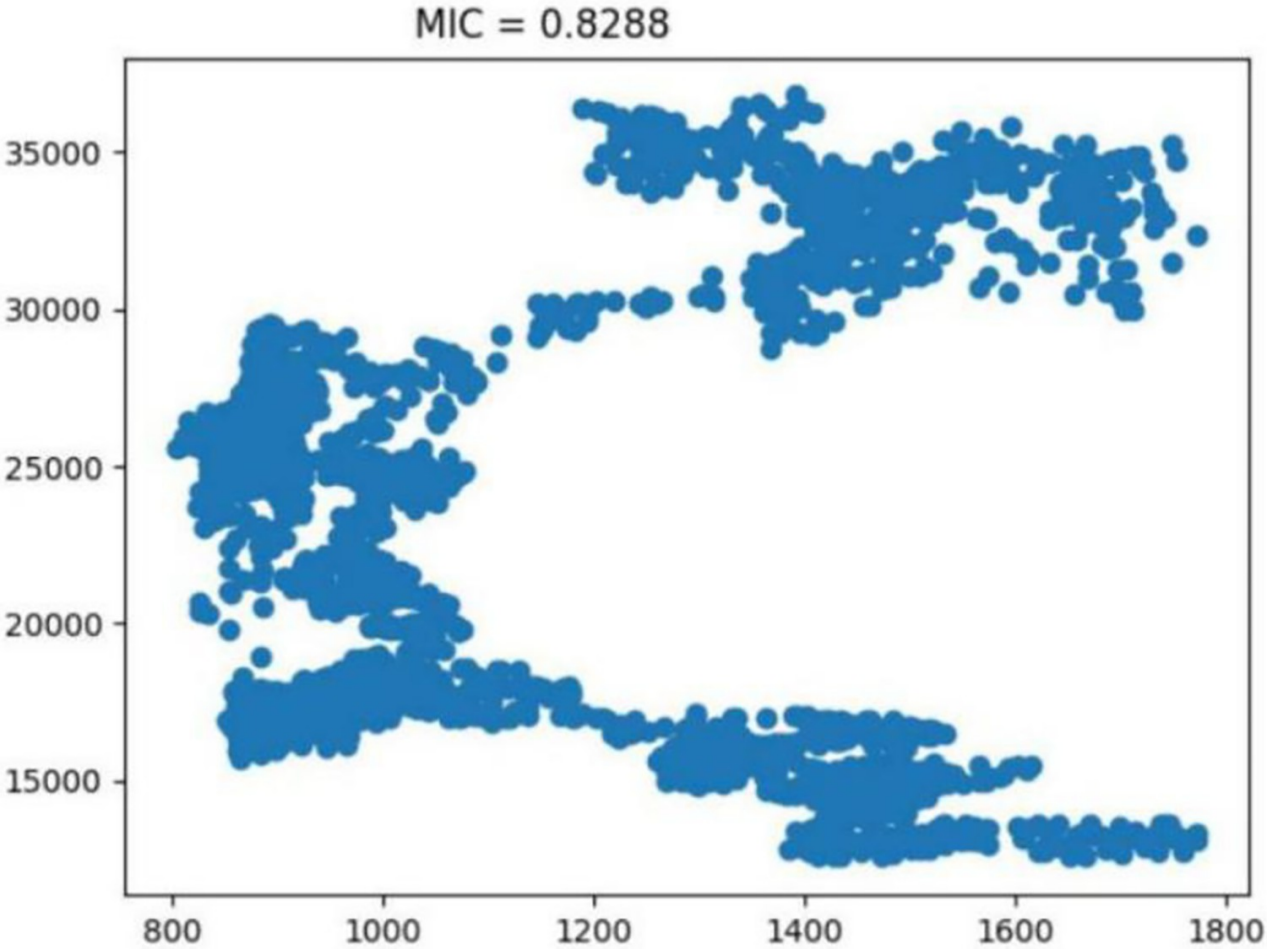
Analysis of the dow jones industrial average indicator. Reprinted from [46] under a CC BY license, with permission from IEEE Access, original copyright 2023.

**Fig 6:**
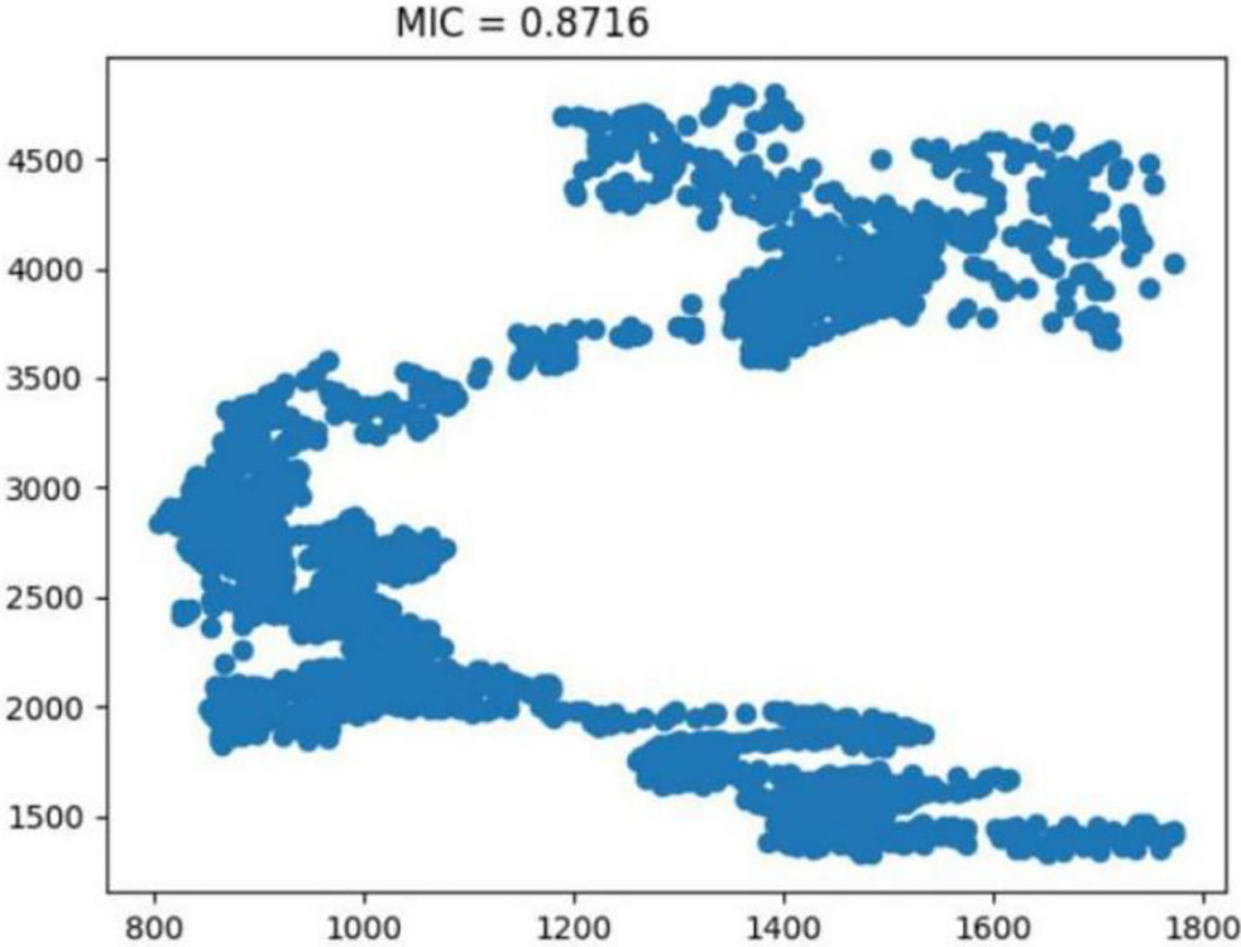
Insights into the standard & poor’s 500 index. Reprinted from [46] under a CC BY license, with permission from IEEE Access, original copyright 2023.

**Fig 7:**
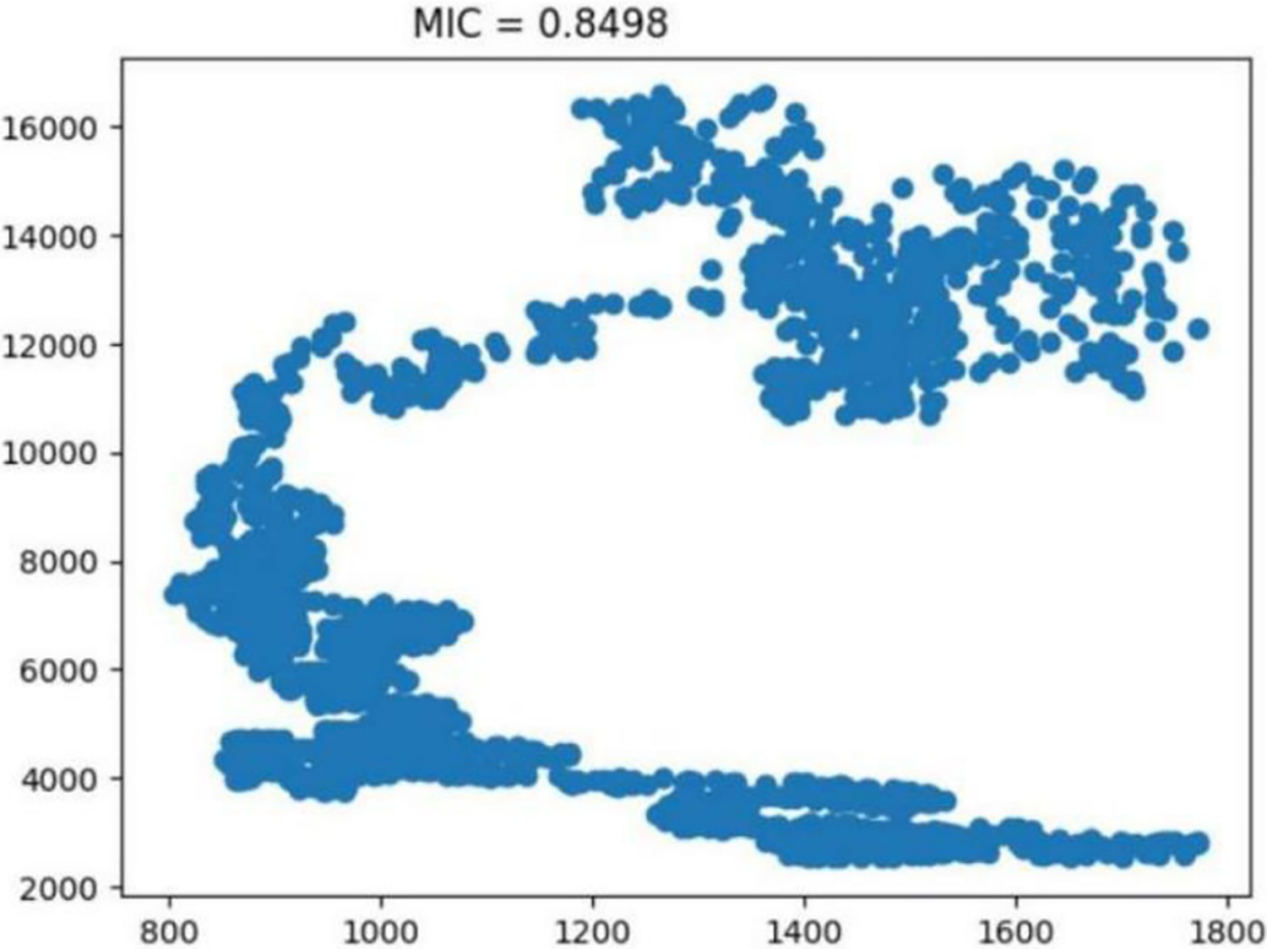
Examination of the NASDAQ composite indicator. Reprinted from [46] under a CC BY license, with permission from IEEE Access, original copyright 2023.

The inaugural segment of our model is the input layer, where we introduce the primary dataset—edamame futures prices. This layer is crucial as it sets the foundation for the model’s data processing. In addition to the primary focus, we incorporate key financial indices such as the Dow Jones Industrial Average (DJIA) (Fig 5), SP 500 (Fig 6), and NASDAQ (Fig 7).

These indices are selected as external influences due to their established impact on market trends and economic indicators. Their inclusion in the input layer allows the model to contextualize the edamame price data within a broader economic landscape, enhancing the accuracy and relevance of our analysis.

Fig 4 visually presents the CIMA-AttGRU model’s architecture, showcasing how each component, from the input layer to the final output, contributes to the processing and analysis of the data. This visualization is instrumental in understanding the intricate workings of the model and how it integrates various elements to provide a comprehensive analysis of edamame futures prices.

The CIMA-AttGRU model’s architecture, as delineated in our study, consists of several layers, each meticulously designed to contribute to the model’s overall predictive analysis. The second tier, the data preprocessing layer, is where the raw input data is refined and prepared for analysis. This includes normalization, handling missing values, and selecting relevant features, all of which are essential for enhancing the accuracy of the predictions.

The third layer is dedicated to CIMA decomposition, where the data is broken down into IMFs. These IMFs represent different frequency components in the data, and their separation is crucial for isolating distinct patterns and trends. In this phase, the AttGRU mechanism is employed to forecast each component independently, allowing for a nuanced analysis of the various elements within the dataset.

Following this, the fifth layer–the combination layer–plays a vital role in bringing together the individual forecasts from each IMF. This consolidation is key to synthesizing the data into a unified analysis, ensuring that all relevant information is factored into the final prediction.

The process culminates in the output layer, the final stage of the model. Here, the aggregated results from the previous layers are scaled to produce the final predictive output. This output embodies the comprehensive prediction derived from the intricate processes of the CIMA-AttGRU model, offering a well-rounded analysis based on the data.

### 3.5. CADA

In time series analysis, there is a challenge of data migration which is a common occurrence in futures markets where changes over time can lead to domain shifts. To address this, we introduce the CADA algorithm. Within the framework of Domain Adversarial Neural Network (DANN) training, initially conceptualized by Ganin and Lempitsky, a pivotal element is the incorporation of the marginal adversarial loss LT [41]. This loss function plays a crucial role in enabling the extraction of domain-invariant feature representations from both the source and target domains. It achieves this through adversarial learning, a process where the model is trained to minimize domain discrepancies, effectively harmonizing the marginal distributions of these domains. However, the conventional DANN model primarily focuses on aligning these domains at the level of their marginal distributions. While this approach has its merits, it can overlook the discriminative structures within the feature space. Such oversight may lead to negative transfer phenomena, where the model inadvertently adopts irrelevant or misleading features from the source domain.

Our research draws upon established matrix optimization-based domain adaptation (DA) techniques to form the foundation of our proposed method. This approach is centered around the estimation of conditional distribution divergences. Specifically, it involves a detailed analysis of the variances in class conditional distributions. The subsequent sections will delve into a more comprehensive discussion on how these estimations are carried out and the implications thereof for the field of domain adaptation.


$$j=e^{u}(X^{u}|Z^{u}),\;c=e^{s}(X^{s}|Z^{s}),$$

$$n=e^{u}(X^{u}|Z^{u}),\;O=e^{s}(X^{s}|Z^{s}),$$

|j−c|∝|n−O| (8)

In the realm of domain adaptation (DA) strategies for DNNs, which are steered by matrix optimization, the task of accurately estimating the equation outlined in Eq 9 is a formidable challenge. This equation integrates both genuine and synthetic labels, and its precise estimation is often complicated by the complexities inherent in batch sampling. These complexities chiefly stem from a potential misalignment between instances in the source and target domains. Such misalignment can skew the accuracy of estimating class-conditional distributions.

Addressing this challenge, our approach introduces a selective mechanism for class subset sampling. This mechanism operates on a principle of alignment congruence; it calculates distribution distances only in instances where there is a match in label spaces between source and target domains during batch sampling. This strategic sampling approach is essential in accurately computing the loss function, which is a critical component of dependent adversarial domain adaptation. It significantly contributes to the model’s robustness and enhances its performance across varied and intricate data environments.

The proposed framework, as articulated in Eq 9, involves a nuanced understanding of data instances, denoted as ZU/S,F, representing the Fth class from either source or target domain. Additionally, OF symbolizes the discriminator assigned to the Fth class. A notable aspect of this framework is the incorporation of pseudo labels from the target domain in each iteration, an approach that significantly augments the model’s precision and adaptability.


$$J(Z)=O(Zp^{U,F}),\;I(Z)=O(Zp^{U,F}),$$

$$O_{J}=OF(J(Z)),\;O_{I}=OF(I(Z)),$$

$$LT^{U}=-\sum_{F=1}^{n}EZp^{U,F}∽D^{U,F}\;\log\;O_{J}$$

$$LT^{S}=-EZp^{U,S}∽D^{S}\;\log\left(1-O_{I}\right)$$

$$LT=LT^{U}+LT^{S}$$ (9)

To address the predictive loss aspect, we introduce a loss function specifically designed for evaluating the accuracy of time series forecasts in agricultural market trends. The predictive loss LP is formulated as follows:

$$LP=\frac{1}{n}\sum_{i=1}^{N}{\left(y_{i}-{\hat{y}}_{i}\right)}^{2}$$ (10)

where N is the number of forecasted points, *y*_*i*_ represents the actual value at time i, and ${\hat{y}}_{i}$ denotes the forecasted value. This loss function, rooted in the MSE, quantifies the deviation of the model’s predictions from the actual market outcomes, thereby guiding the optimization process towards more accurate forecasts.

Incorporating LP into our domain adaptation framework, the overall objective be- comes the minimization of both domain adaptation loss and predictive loss. This dual- objective approach ensures that our model not only learns domain-invariant features but also hones its forecasting accuracy, making it particularly well-suited for predicting agricultural market trends with high precision and reliability.

In conclusion, our CADA method extends the conventional DANN framework by incorporating a predictive loss component. This integration enhances the model’s capability to adapt across domains while simultaneously improving its forecasting accuracy, offering a robust solution for domain adaptation challenges in the context of agricultural market trend forecasting.

## 4. Experimental design and methodology

### 4.1. Configuration of experimental setup

The study was conducted using a computational setup comprised of a Windows 10 operating system. The hardware included an Intel(R) Core (TM) i7 CPU, an NVIDIA GTX4090 graphics card, and 24.00 GB of RAM. For software, Python 3.9 served as the programming language, with PyCharm utilized as the development environment.

### 4.2. Data acquisition and analytical approach

Data for this analysis was sourced from the Tushare platform, an open-access repository for financial big data. The study focused on key financial indices, U.S. Dollar Index (USDX), and NASDAQ. To assess the interrelations among these indices, the Maximum Information Coefficient (MaxIC) was employed in Table 2. This MaxIC is particularly adept at handling nonlinear data relationships.

**Table 2:** Outcome analysis of MIC computation.

Data	NASDAQ	S&P 500	SSE	USDX	DJIA
MIC	0.85	0.87	0.44	0.46	0.83

For a pair of variables (A, B), the methodology involves partitioning them into p and q intervals along their respective axes, thereby creating a grid of pq cells. Given a dataset of these two-dimensional variables, the MaxIC is computed as follows:

$$Min{Val}_{p,q}=\min\left\{p,q\right\},$$

$$\log\;Calc=\log_{2}\;Min{Val}_{p,q},$$

$$MCalc{\left(Dataset\right)}_{p,q}=\frac{Max{Info}^{*}(Dataset,p,q)}{\log\;Calc},$$

$$Limit=Func{\left(SampleSize\right)}^{\left\{MCalc{\left(Dataset\right)}_{p,q}\right\}},$$

$${MaxIC}_{MaxIC}(A,B)=\begin{array}{c} max\\ pq<Limit \end{array}$$ (11)

In this formula, MaxIC_MaxIC_(*A*,*B*) signifies the maximum information coefficient for the variables (A, B). The term MaxInfo*(*Dataset*,*p*,*q*) represents the maximum mutual information obtainable from the dataset for the given partitioning. The SampleSize denotes the total number of data points, with Func(*SampleSize*) being a function of this size, typically set to *SampleSize*^0.6^ [45].

In this study, we conducted a comprehensive analysis using a dataset encompassing the edamame futures trading activities on the Dalian Commodity Exchange. The dataset is comprised of a total of 2750 entries. These entries were strategically segmented into different subsets for the purpose of the experiment: 2200 entries were allocated for the training set, while 275 entries each were designated for the verification and test sets, respectively. This distribution was meticulously planned to ensure a robust and thorough evaluation of the dataset.

The data used in this study was sourced from the Tushare platform, an open-access repository for financial big data. Our analysis focused on key financial indices, including the U.S. Dollar Index (USDX) and NASDAQ. These indices were selected due to their established impact on market trends and economic indicators, providing important context for understanding the dynamics of the edamame futures market. The primary dataset comprised edamame futures trading activities on the Dalian Commodity Exchange, totaling 2,750 data entries. The choice to focus on edamame futures was driven by the significant economic impact and high market price volatility associated with this commodity. To ensure a robust and comprehensive evaluation, we employed a carefully designed data segmentation strategy: 2,200 entries were allocated to the training set, while 275 entries each were designated for the validation and test sets. This approach allows for a thorough assessment of the dataset and enhances the reliability of the experimental results. By incorporating these key financial indices as external influences and focusing on the edamame futures price data, our study aims to contextualize the analysis within a broader economic landscape. This approach enhances the accuracy and relevance of our findings, providing valuable insights into the complex dynamics of the edamame futures market.

### 4.3. Data preprocessing and normalization techniques

The adopted methodology for data standardization is encapsulated by the following mathematical representation:

$${Sum}_{v,t}=V_{t-1}+V_{t-2}$$

$$V_{t}=\frac{{Sum}_{v,t}}{2}$$ (12)

In this equation, Vt signifies the interpolated value at time t, while Vt−1 and Vt−2 represent the recorded data from the immediate and second preceding trading days at time t, respectively. Subsequent to this interpolation, a comprehensive integration of the influencing factors and the edamame futures data is conducted.

Given the substantial variance often present in the magnitudes of different features within the dataset, there is a potential risk of inadequate learning by the algorithm. This approach normalizes the data, scaling the features to a uniform range of [0, 1], thereby enhancing algorithmic learning efficiency.

### 4.4. Construction of time series data

**Fig 8:**
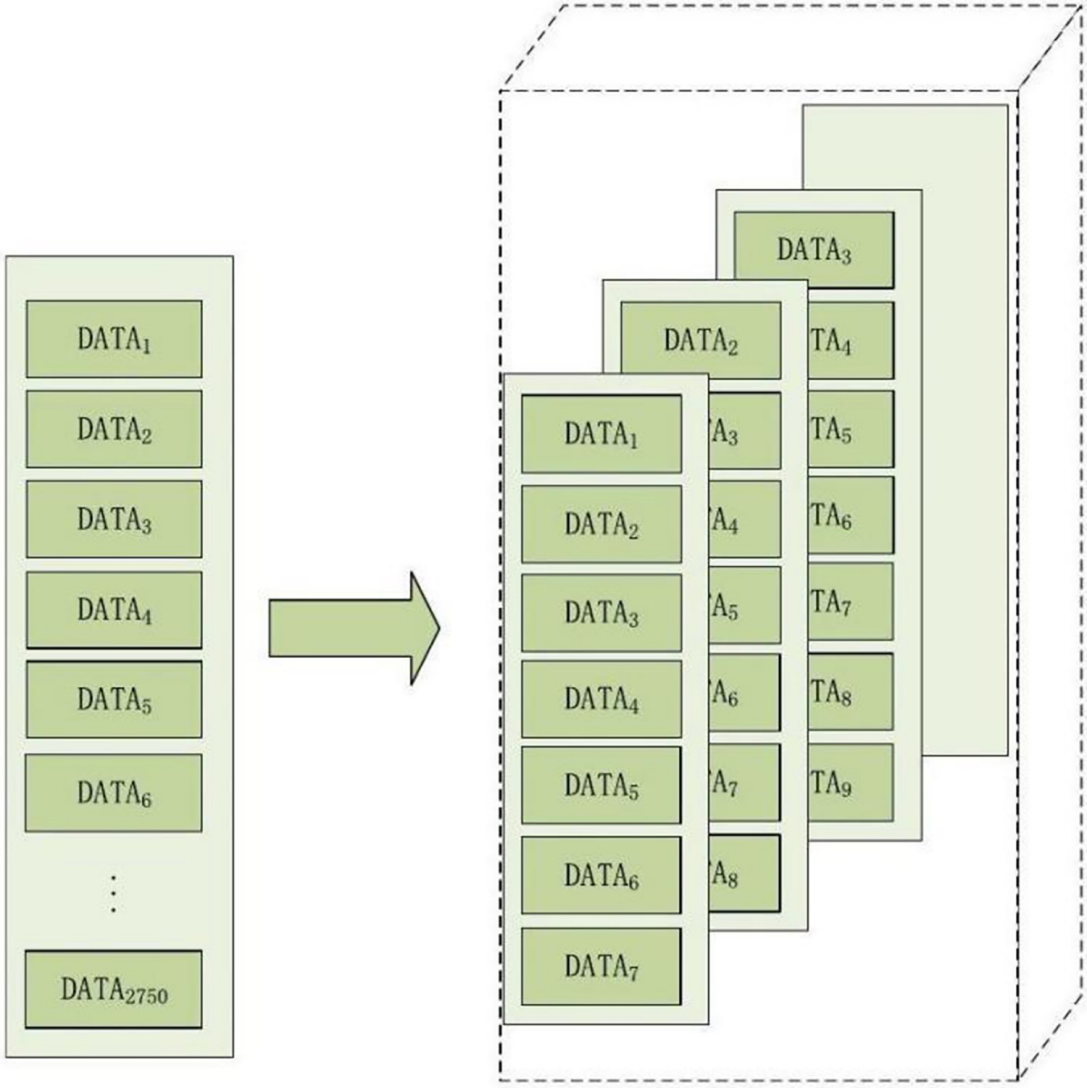
Procedure for building time series sequences.

The experimental design, as depicted in Fig 8, incorporates a step size of 1 and a sequence length of 7. With a total data length of 2750, the data is restructured into a three-dimensional format, yielding a final dimension of (2744, 7, 12). This reconfiguration involves grouping the data into segments of seven rows and eight columns, starting from x − 6 data points.

### 4.5. Optimization and validation of model parameters

The efficacy of neural networks in generalizing performance often surpasses that of linear models, attributed to their intricate parameter architecture. However, this complexity necessitates meticulous fine-tuning of numerous hyperparameters to achieve optimal generalization capabilities. In this experimental framework, an initial range for the optimal hyperparameters was established based on prior experiential knowledge. We conducted Bayesian optimization privately to search for optimal hyperparameters. However, we discovered that extreme hyperparameter optimization could lead to a reduction in the model’s generalization performance. While the chosen reasonable hyperparameter values based on experience may not yield the best results on our specific dataset, they help ensure that the models are less sensitive to parameter variations, promoting better generalization and ease of fine-tuning. Regarding the choice of 32 units in Table 3, this value was selected based on our prior experience and considering the trade-off between model complexity and computational efficiency. We acknowledge that exploring a wider range of unit values could potentially improve performance. However, our primary focus was on maintaining consistency across models to enable a fair comparison. We believe that the chosen hyperparameter values strike a balance between model performance and generalization ability.

**Table 3:** Parameters governing model configuration.

Model	Batch Size	Other Parameters	Activation	Epochs	Units
LSTM [27]	32	default	tanh	100	32
SVR [29]	-	kernel = ‘rbf’, C = 1.0,	-	-	-
		epsilon = 0.2			
GRU [28]	32	default	tanh	100	32
AttGRU [30]	32	default	sigmoid	100	32
RainDrop [31]	32	default	sigmoid	100	32
SimTSC [32]	32	default	sigmoid	100	32
LB-SimTSC [33]	32	default	sigmoid	100	32
TodayNet [34]	32	default	sigmoid	100	32
Attention-GRU [35]	32	filters = 32, kernel size = 1, pool size = 1	tanh	100	32
			sigmoid		
Attention-LSTM [36]	32	filters = 32, kernel size = 1, pool size = 1	-	100	32
			tanh		
Attention-AttGRU [37]	32	filters = 32, kernel size = 1, pool size = 1	sigmoid	100	32
		activation2 = ‘leaky relu’	multiple		

In addition to the hyperparameter optimization techniques discussed earlier, we also incorporated early stopping criteria into our model training process to further enhance the model’s generalization ability and prevent overfitting. Early stopping is a widely used regularization technique in machine learning that involves monitoring the model’s performance on a validation set during training and stopping the training process when the performance on the validation set starts to degrade. To implement early stopping, we split our data into training, validation, and test sets. During the training process, we monitored the model’s performance on the validation set at each epoch. If the model’s performance on the validation set did not improve for a specified number of epochs (defined as the patience parameter), the training was stopped, and the model weights from the best-performing epoch were retained. This approach allowed us to determine the optimal value for the patience parameter that maximized the model’s performance on the validation set while preventing overfitting. The incorporation of early stopping criteria into our model training process not only improved the model’s performance but also provided a more rigorous and comprehensive analysis of our approach. By leveraging this technique, we ensured that our proposed CIMA-AttGRU model is robust, reliable, and capable of generalizing well to unseen data.

## 5. Experimental analysis and model comparison

**Fig 9:**
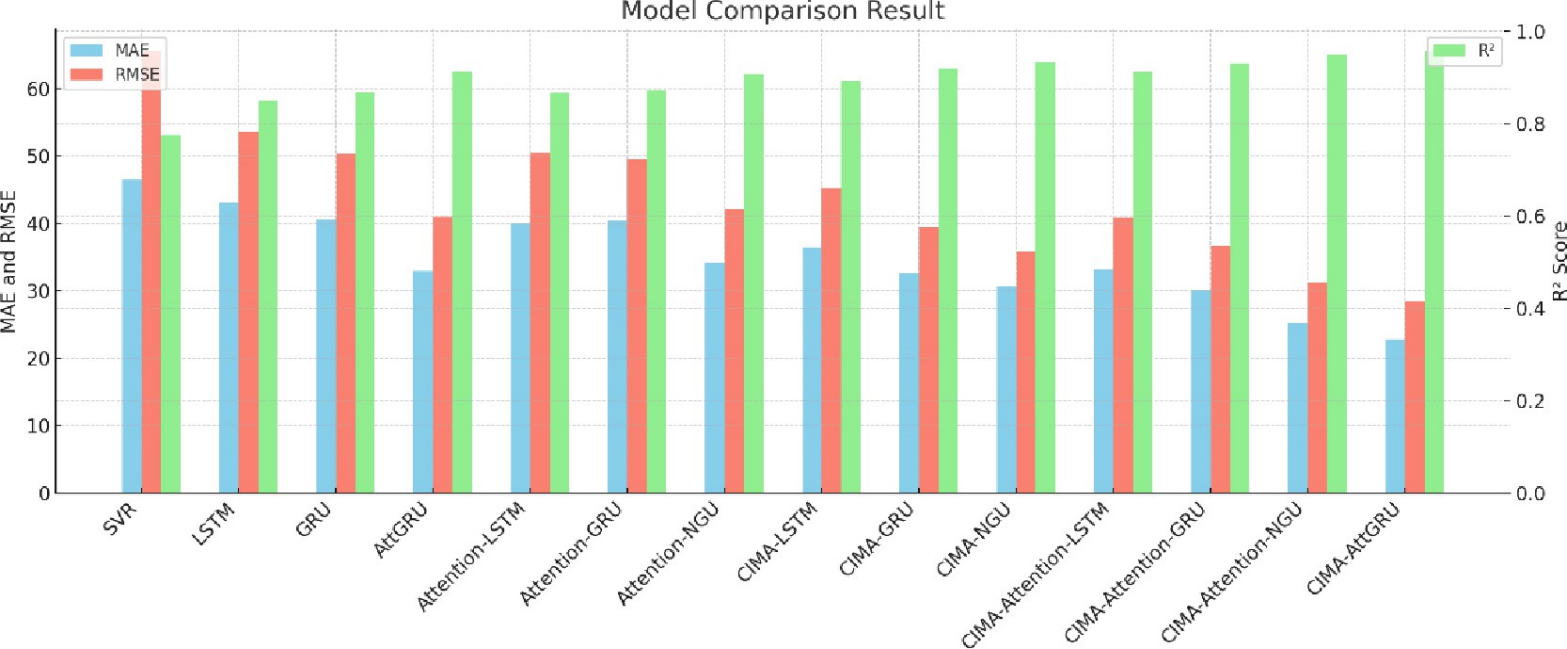
Outcome of comparative analysis between models.

The performance of these models was rigorously evaluated using metrics such as RMSE, MAE (Mean Absolute Error), and R2, seen in Fig 9. The results, detailed in Table 4, were derived using the test dataset. These results highlighted that the conventional regression model SVR exhibited the least predictive accuracy. Within the realm of deep learning models, AttGRU demonstrated superior performance over Attention-GRU, with CIMA-AttGRU emerging as the most effective predictive model.

**Table 4:** Ablation study results comparing different configurations of the model.

Model Configuration	MAE	RMSE
Base Model (GRU)	0.06	0.09
CIMA + GRU	0.05	0.07
GRU + AttGRU	0.04	0.06
CIMA-AttGRU (Full Model)	**0.03**	**0.04**

A comparative analysis, as evidenced in Table 4, reveals that the AttGRU model achieved a significant improvement in prediction accuracy compared to the standard GRU model. This improvement is quantified by a reduction in MAE by 7.47, a decrease in RMSE by 8.72. Similarly, when comparing the CIMA-enhanced models, CIMA-AttGRU outperformed CIMA-Attention-GRU, evidenced by a reduction in MAE by 7.41, a decrease in RMSE by 8.10.

**Fig 10:**
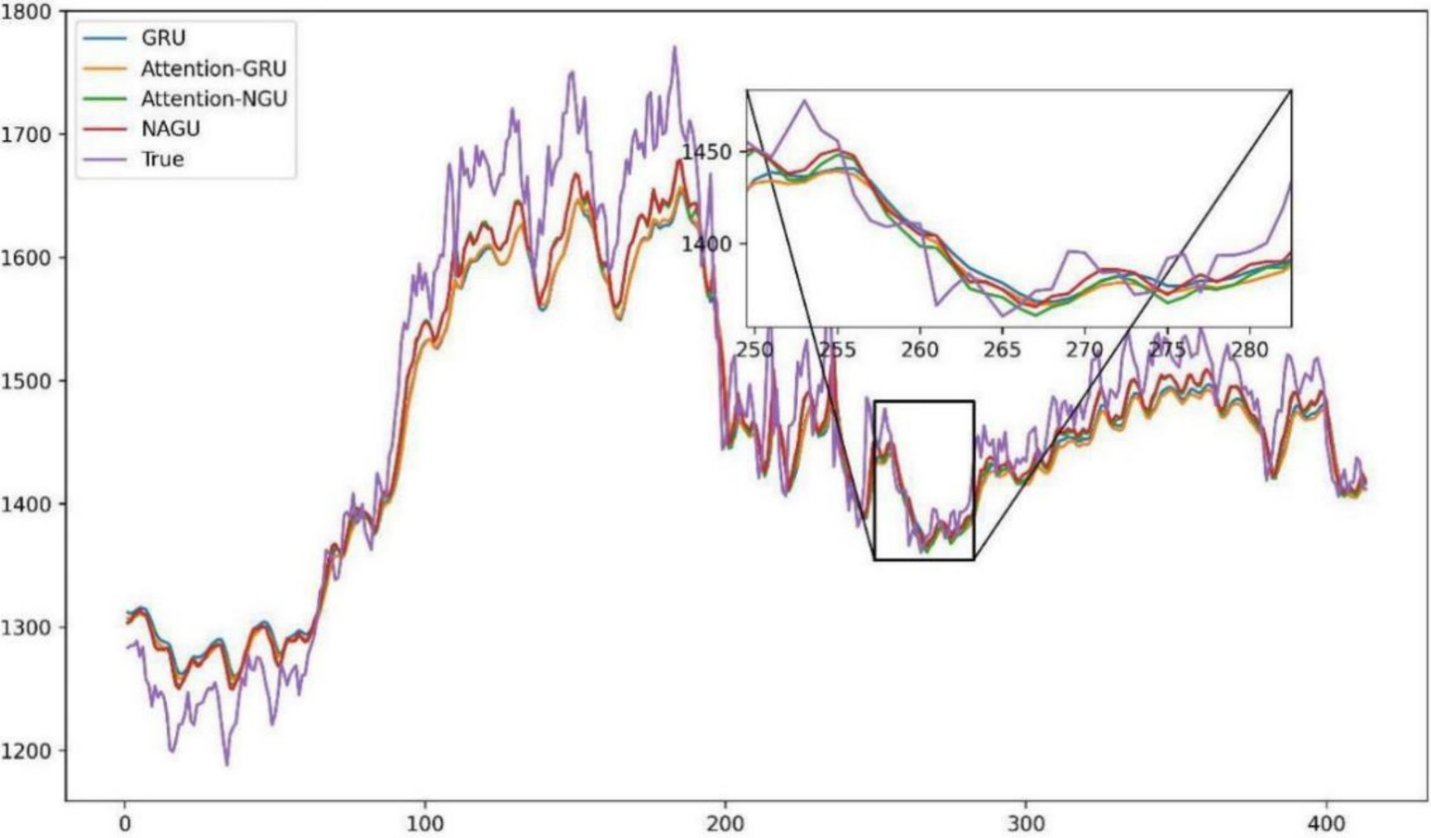
Comparative analysis of forecasted and actual values in GRU, Attention-GRU, Attention- NGU, and AttGRU models.

The comparative visualization of predicted versus actual values for GRU, Attention- GRU, Attention-NGU, AttGRU, CIMA-Attention-GRU, and CIMA-AttGRU models is provided in Fig 10. This graphical representation underscores the enhanced predictive capabilities of the CIMA-AttGRU model over its counterparts.

**Fig 11:**
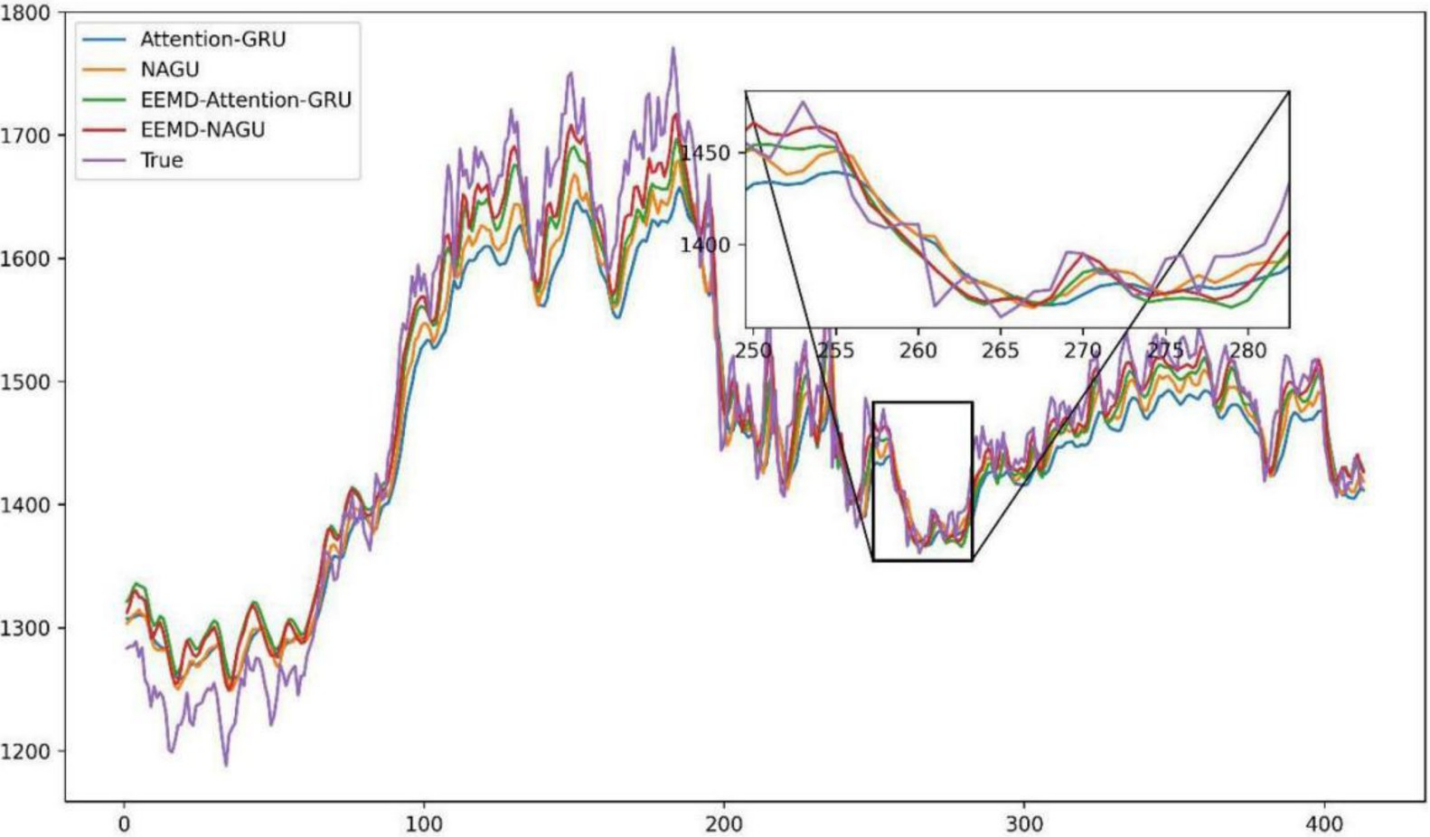
Forecasted versus actual outcomes in Attention-GRU, AttGRU, CIMA-Attention-GRU, and CIMA-AttGRU models.

To assess the individual contributions of the CIMA and AttGRU components to the forecasting performance of the CIMA-AttGRU model, we designed a series of ablation experiments depicted in Fig 11. These experiments were structured to incrementally remove key components from the full model and evaluate the resulting impact on prediction accuracy. The experiments were conducted on a dataset comprising edamame futures prices, with the following configurations for model evaluation:

- **Base Model (GRU)**: A generic GRU model without CIMA preprocessing or attention mechanisms.
- **CIMA + GRU**: Incorporation of CIMA for data preprocessing, followed by a generic GRU for time series forecasting.
- **GRU + AttGRU**: A hybrid model utilizing generic GRU layers, supplemented by AttGRU layers to introduce attention mechanisms.
- **CIMA-AttGRU (Full Model)**: The complete model incorporating both CIMA preprocessing and AttGRU layers.

The outcomes of the ablation study are summarized in Table 4, highlighting the performance metrics across different model configurations.

The ablation study reveals the critical role played by both CIMA and AttGRU in enhancing the forecasting performance of the model. While the base GRU model provides a baseline for comparison, the addition of CIMA preprocessing significantly reduces both MAE and RMSE, indicating the importance of noise reduction and signal decomposition in improving prediction accuracy. The incorporation of the attention mechanism via AttGRU layers further improves the model’s ability to capture temporal dependencies, as evidenced by the reduced error metrics.

The full CIMA-AttGRU model, combining both CIMA preprocessing and AttGRU, achieves the best performance across all metrics. This underscores the synergy between CIMA’s ability to prepare the data through signal decomposition and the AttGRU’s capabiity to focus on relevant temporal features, leading to more accurate predictions of edamame futures prices.

## 6. Statistical significance tests for model performance

To rigorously evaluate the predictive performance of the CIMA-AttGRU model relative to other models, we employed the Diebold-Mariano (DM) test [46] to compare the statistical significance of differences in prediction accuracy. The DM test is a widely used non-parametric statistical test for comparing the forecast errors of two models. The null hypothesis is that there is no significant difference in the predictive accuracy of the two models. If the p-value is less than the significance level (e.g., 0.05), the null hypothesis can be rejected, indicating a significant difference in the models’ performance. We selected six main benchmark models (SVR, LSTM, GRU, Attention-LSTM, Attention-GRU, AttGRU, Attention-AttGRU, and CIMA-Attention-GRU) and conducted pairwise comparisons with the CIMA-AttGRU model. The test results are presented in Table 5. As shown, the CIMA-AttGRU model demonstrates statistically significant differences in predictive accuracy compared to all other models (p < 0.05). The absolute values of the DM statistics and the magnitude of the p-values are consistent with the performance metrics in Table 4, indicating that models with larger performance gaps have larger absolute DM statistics and smaller corresponding p-values.

**Table 5:** Statistical significance tests for model performance.

Model	DM Statistic	p-value
SVR	-8.21	<0.001
LSTM	-5.79	<0.001
GRU	-4.34	<0.001
Attention-LSTM	-3.96	<0.001
Attention-GRU	-3.13	0.002**
AttGRU	-2.49	0.013*
Attention-AttGRU	-2.15	0.031*
CIMA-AttGRU (Full Model)	**-1.96**	0.049*

Note: *, **, and *** denote significance at the 0.05, 0.01, and 0.001 levels, respectively.

The DM test results provide strong evidence supporting the superior performance of the CIMA-AttGRU model in the task of soybean futures price prediction. The integration of CIMA decomposition and attention mechanism enables the model to better capture the intrinsic patterns in the data and enhance the accuracy of predictions. These findings are consistent with the experimental results presented throughout the paper, reinforcing the effectiveness of the proposed model. The statistical significance tests add robustness to our claims about the CIMA-AttGRU model’s performance and strengthen the overall credibility of the research. By demonstrating the model’s statistically significant improvements over a range of benchmark models, we provide compelling evidence for its practical value in forecasting soybean futures prices.

## 7. Discussion

The experimental results demonstrate the superior performance of the CIMA-AttGRU model in predicting soybean futures prices compared to other benchmark models. This can be attributed to its unique architecture, which combines CIMA for noise reduction and AttGRU for capturing dynamic temporal dependencies. CIMA enables the model to filter out market noise and decompose the time series data into intrinsic modes, addressing the high volatility in agricultural markets. This allows the model to focus on essential features and underlying patterns, leading to improved prediction accuracy.

The AttGRU component, with its attention mechanism, dynamically adjusts to temporal dependencies, enabling the model to capture nuanced fluctuations in futures prices often overlooked by traditional models. The attention mechanism helps prioritize relevant information and adapt to changing market conditions. The incorporation of CADA enhances the model’s robustness by addressing domain adaptivity, which is crucial for soybean futures, where price determinants vary significantly over time and across regions. CADA aligns feature distributions between source and target domains, improving generalization ability and adaptability to new market conditions. The ablation study highlights the individual contributions of CIMA and AttGRU components to the overall performance, showing their crucial roles in improving prediction accuracy. While CIMA-AttGRU demonstrates significant improvements in prediction accuracy, its increased complexity may lead to longer training times and higher computational requirements. Future research could explore ways to optimize the model’s architecture and training process to improve efficiency without compromising predictive performance.

## 8. Conclusions

In this study, we propose the CIMA-AttGRU model for predicting soybean futures prices. The model combines CIMA for noise reduction and AttGRU for capturing temporal dependencies, achieving superior performance compared to benchmark models. The experimental results, supported by statistical tests, demonstrate the effectiveness of CIMA-AttGRU in capturing the complex dynamics of the soybean futures market and providing accurate predictions. The model’s ability to filter out noise, focus on relevant features, and adapt to changing market conditions makes it valuable for stakeholders in the soybean industry. The proposed architecture can be adapted to other agricultural commodities and financial markets, where accurate forecasting is crucial. Future research directions include incorporating additional external factors and exploring the integration of CIMA-AttGRU with other machine learning techniques to improve its generalization ability and adaptability. In conclusion, CIMA-AttGRU represents a significant advancement in agricultural market forecasting, providing a powerful tool for predicting soybean futures prices. Its superior performance, robustness, and adaptability contribute to the growing body of research on the application of advanced machine learning techniques in the financial and agricultural sectors.
